# Supplementary material for: Furosine Posed Toxic Effects on Primary Sertoli Cells through Regulating Cep55/NF-κB/PI3K/Akt/FOX01/TNF-α Pathway
Source: Int J Mol Sci. 2019 Jul 30;20(15):3716. doi: 10.3390/ijms20153716 (PMC6696181; doi:10.3390/ijms20153716)
Supplement: Supplementary file 1 [file ijms-20-03716-s001.pdf]

Table S1. The upregulated chemicals in testicular tissue

	Special upregulated metabolites
1	DG(18:1(9Z)/18:3(9Z,12Z,15Z)/0:0)[iso2]
2	DG(16:0/18:3(9Z,12Z,15Z)/0:0)[iso2]
3	DG(18:0/18:3(9Z,12Z,15Z)/0:0)[iso2]
4	TG(15:1(9Z)/17:0/18:2(9Z,12Z))[iso6]
5	DG(16:1(9Z)/18:1(9Z)/0:0)[iso2]
6	DG(O-16:0/18:1(9Z))
7	TG(15:0/17:1(9Z)/20:3(8Z,11Z,14Z))[iso6]
8	DG(16:0/18:1(9Z)/0:0)
9	TG(16:0/16:1(9Z)/18:1(9Z))[iso6]
10	TG(13:0/19:1(9Z)/22:3(10Z,13Z,16Z))[iso6]
11	DG(16:0/16:1(9Z)/0:0)[iso2]
12	DG(16:0/16:0/0:0)
13	PC(20:4(5Z,8Z,11Z,14Z)/20:4(5Z,8Z,11Z,14Z))
14	TG(16:1(9Z)/18:2(9Z,12Z)/18:2(9Z,12Z))[iso3]
15	TG(16:1(9Z)/16:1(9Z)/18:2(9Z,12Z))[iso3]
16	PG(22:4(7Z,10Z,13Z,16Z)/22:6(4Z,7Z,10Z,13Z,16Z,19Z))
17	TG(17:2(9Z,12Z)/20:5(5Z,8Z,11Z,14Z,17Z)/22:6(4Z,7Z,10Z,13Z,16Z,19Z))[iso6]
18	PG(22:6(4Z,7Z,10Z,13Z,16Z,19Z)/20:2(11Z,14Z))
19	TG(16:1(9Z)/18:2(9Z,12Z)/20:3(8Z,11Z,14Z))[iso6]
20	TG(16:1(9Z)/17:1(9Z)/19:0)[iso6]
21	SM(d18:1/16:0)
22	PS(22:4(7Z,10Z,13Z,16Z)/20:0)
23	TG(13:0/19:1(9Z)/20:3(8Z,11Z,14Z))[iso6]
24	PC(22:6(4Z,7Z,10Z,13Z,16Z,19Z)/16:0)
25	PE-Cer(d16:1(4E)/20:0)
26	PC(20:4(8E,11E,14E,17E)/16:0)
27	PnE(16:0/18:1(9Z))
28	PA(22:4(7Z,10Z,13Z,16Z)/19:0)
29	CL(1'-[20:4(5Z,8Z,11Z,14Z)/20:0],3'-[20:0/18:2(9Z,12Z)])
30	PE(O-16:0/22:6(4Z,7Z,10Z,13Z,16Z,19Z))
31	PE(O-16:0/22:5(4Z,7Z,10Z,13Z,16Z))
32	PE(16:0/20:4(5Z,8Z,11Z,14Z))
33	PE(18:0/22:4(7Z,10Z,13Z,16Z))
34	PE(O-16:0/18:3(6Z,9Z,12Z))
35	PE(18:0/16:1(9Z))
36	PE(21:0/18:3(9Z,12Z,15Z))
37	PE(P-16:0/22:6(4Z,7Z,10Z,13Z,16Z,19Z))

Table S2. The downregulated chemicals in testicular tissue

	Special downregulated metabolites
1	Dinoravicholic acid
2	8,15-DiHETE
3	(7Z)-14-hydroxy-10,13-dioxoheptadec-7-enoic acid
4	17-hydroxy-1-oxo-2,3-seco-androstan-3-oic acid
5	methyl 8-[2-(2-formyl-vinyl)-3-hydroxy-5-oxo-cyclopentyl]-octanoate
6	Prosafrinine
7	docebenone
8	Panaquinquecol 7
9	DG(15:1(9Z)/17:0/0:0)[iso2]
